# Clinical implications of septic cardiomyopathy: A narrative review

**DOI:** 10.1097/MD.0000000000037940

**Published:** 2024-04-26

**Authors:** Hiroaki Hiraiwa, Daisuke Kasugai, Takahiro Okumura, Toyoaki Murohara

**Affiliations:** a Department of Cardiology, Nagoya University Graduate School of Medicine, Nagoya, Japan; b Department of Emergency and Critical Care Medicine, Nagoya University Graduate School of Medicine, Nagoya, Japan.

**Keywords:** diagnosis, sepsis, septic cardiomyopathy, septic shock, treatment

## Abstract

Sepsis is caused by the body’s dysregulated response to infection, which can lead to multiorgan injury and death. Patients with sepsis may develop acute cardiac dysfunction, termed septic cardiomyopathy, which is a global but reversible dysfunction of both sides of the heart. This narrative review discusses the mechanistic changes in the heart during septic cardiomyopathy, its diagnosis, existing treatment options regarding severity and course, and emerging treatment approaches. Although no standardized definition for septic cardiomyopathy exists, it is described as a reversible myocardial dysfunction that typically resolves within 7 to 10 days. Septic cardiomyopathy is often diagnosed based on electrocardiography, cardiac magnetic resonance imaging, biomarkers, and direct invasive and noninvasive measures of cardiac output. Presently, the treatment of septic cardiomyopathy is similar to that of sepsis, primarily focusing on acute interventions. Treatments for cardiomyopathy often include angiotensin-converting enzyme inhibitors, angiotensin receptor blockers, and diuretics. However, because of profound hypotension in sepsis, many cardiomyopathy treatments are contraindicated in patients with septic cardiomyopathy. Substantial efforts have been made to study the pathophysiological mechanisms and diagnostic options; however, the lack of a uniform definition for septic cardiomyopathy is challenging for physicians when considering treatments. Another challenge for physicians is that the treatment for septic cardiomyopathy has only focused on acute intervention, whereas the treatment for other cardiomyopathies has been provided on a long-term basis. A better understanding of the underlying mechanisms of septic cardiomyopathy may contribute to the development of a unified definition of the condition and novel treatment options.

## 1. Introduction

Sepsis is a clinical syndrome characterized by widespread physiological and biochemical abnormalities caused by a dysregulated response to infection, which can cause multiorgan injuries and death.^[1,2]^ Septic shock is a severe complication of sepsis that is associated with vasodilation and organ failure.^[1]^ In 2017, approximately 48.9 million sepsis cases and 11 million sepsis-related deaths were recorded worldwide.^[3]^ Patients with sepsis may develop acute cardiac dysfunction, termed septic cardiomyopathy, which is an increasingly recognized form of transient cardiac dysfunction in these individuals. The prevalence of septic cardiomyopathy in patients with sepsis ranges 10% to 70%.^[4]^ A large-scale retrospective study reported that the incidence of septic cardiomyopathy is 28.2%.^[5]^

Notably, cardiomyopathy in the presence of systemic infection differs from general cardiomyopathy, which occurs without sepsis. Cardiomyopathies include signs and symptoms common in heart failure, such as reduced ejection fraction along with peripheral edema, fatigue, dyspnea on exertion, presyncope, syncope, and cardiac ischemia.^[6]^ In patients who survive episodes, septic cardiomyopathy is unique in its reversibility within 7 to 10 days.^[7]^

Despite extensive efforts to study the clinical presentation, pathophysiological mechanisms, and diagnostic options, there is no uniform definition for septic cardiomyopathy.^[8,9]^ Several researchers have suggested that septic cardiomyopathy can be defined as a global but reversible dysfunction of both sides of the heart induced by sepsis.^[10,11]^

Researchers and clinicians have identified several fundamental features of septic cardiomyopathy. For example, a hallmark of septic cardiomyopathy is its reversibility,^[9,12]^ and studies have shown that patients who survive recover full cardiac function within 7 to 10 days after sepsis is resolved.^[13]^ Nevertheless, it is a life-threatening condition, and its presence in septic shock reportedly increases the mortality rate in patients by 70% to 90%.^[12]^ Furthermore, the severity of sepsis, characterized by the degree of biochemical changes (e.g., inflammatory markers and white blood cell count), level of treatment with vasoactive substances needed, and extent of multiorgan damage, can influence its reversibility and long-lasting effects.^[1]^ The mechanistic understanding of septic cardiomyopathy has changed largely because of advances in molecular biology techniques, influencing diagnostic and therapeutic approaches.

This narrative review focuses on the mechanisms underlying septic cardiomyopathy, its diagnosis, existing treatment options regarding its severity and course, and emerging approaches to treatment.

## 2. Methods

We conducted a literature search using PubMed/MEDLINE, Scopus, and Web of Science from inception to December 2022 for studies that assessed septic cardiomyopathy. Our search included the following Medical Subject Headings terms: “septic cardiomyopathy” OR “sepsis” OR “septic shock” AND “diagnosis” AND “treatment” AND “cardiac function.” The search yielded more than 1000 results. Duplicated studies, studies with unrelated topics, inappropriate study design, inaccessible full text, and incomplete data were excluded. Finally, 88 articles were included and cited in this review.

## 3. Discussions/observations

### 3.1. Pathophysiology of septic cardiomyopathy

**Figure 1.**
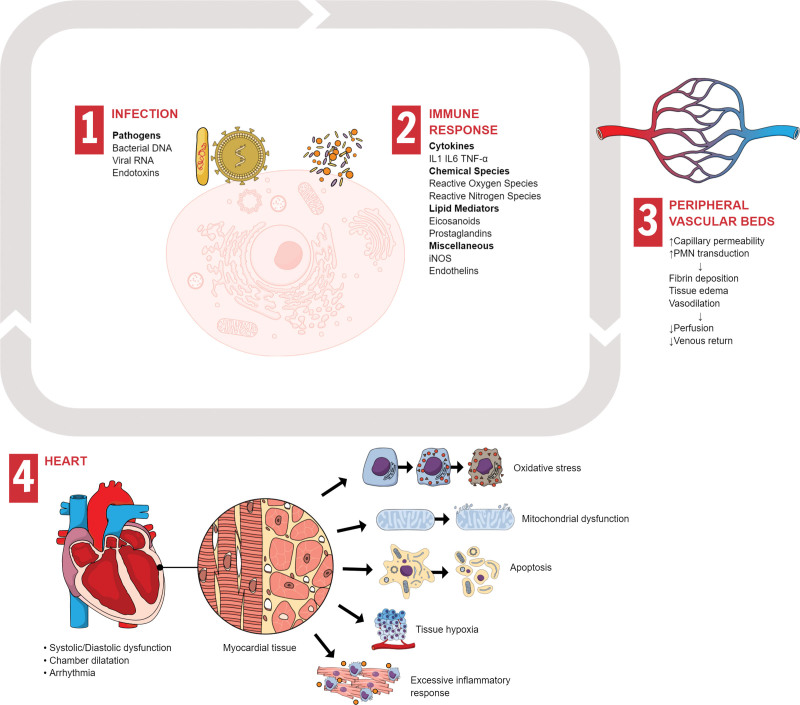
Pathophysiology and mechanisms of septic myocardial dysfunction. Synopsis of basic pathophysiology and mechanisms of sepsis-induced cardiomyopathy: Septic cardiomyopathy can be caused by several factors, including the release of inflammatory mediators and direct toxic effects of bacteria and their toxins on the myocardium. Additionally, the body’s immune response can cause septic cardiomyopathy, leading to inflammation and damage to the heart muscle. Peripheral vascular beds can become constricted in septic cardiomyopathy, which may be caused by the body’s response to sepsis, causing the release of various mediators, such as catecholamines and cytokines. Restriction of blood flow to the peripheral tissues can lead to organ ischemia, hypoperfusion, and organ failure. Blood vessels may leak, allowing the fluid to escape into the surrounding tissue. This can lead to edema in the peripheral tissues and organs. Myocardial dysfunction can occur in multiple ways during sepsis. For example, the left and/or right ventricles can become impaired during systole or diastole, with or without inadequate cardiac output and oxygen delivery. IL = interleukin, iNOS = induced nitric oxide synthase, PMN = polymorphonuclear cell, TNF = tumor necrosis factor.

Septic cardiomyopathy is believed to be caused by myocardial ischemia resulting from inadequate blood flow and activation of the immune system via chemical mediators, such as endotoxins, cytokines, and nitric oxide.^[4,8,13,14]^ The pathophysiology and mechanisms underlying septic cardiomyopathy are illustrated in Figure 1.

Numerous bacterial toxins and other mediators are involved in sepsis pathogenesis. Parrillo et al^[15]^ first hypothesized that a circulating myocardial depressant affects ventricular function in sepsis. Several factors responsible for sepsis have been identified and reviewed previously.^[16,17]^ Septic cardiomyopathy may result from an imbalance in the expression of pro- and anti-inflammatory cytokines.^[18]^ Overproduction of proinflammatory cytokines, such as interleukin-6 (IL-6) and tumor necrosis factor-alpha (TNF-alpha), is crucial in cardiomyocyte apoptosis and injury.^[2]^ Notably, TNF-alpha and IL-1-beta act synergistically to cause myocardial depression.^[19]^ TNF-alpha and IL-1-beta induce the release of additional factors, including nitric oxide, that alter myocardial function.^[20]^ Another cytokine, IL-6, was upregulated in patients with severe coronavirus disease and myocardial inflammation.^[21]^

Toll-like receptors (TLRs) are a class of proteins that play a key role in the innate immune system.^[22]^ In the context of septic cardiomyopathy, TLRs could provide a key link between septic cardiomyopathy and the immune system. TLRs have been implicated in several ways. For instance, TLR4 is expressed in a variety of cells, including cardiomyocytes.^[23]^ TLR4 increases the expression of inflammatory cytokines, including TNF-alpha, IL-1 beta, and IL-6, which may lead to myocardial depression.^[24]^ TLR7 has been found to provide cardioprotection in septic conditions.^[25]^

The complement activation product C5a is associated with septic cardiomyopathy.^[26]^ The complement system is activated during sepsis, leading to an increase in C5a levels, which then interact with its receptors on cardiomyocytes. The binding of C5a to two G-protein coupled receptors, C5aR1 and C5aR2, triggers a significant increase in reactive oxygen species and intracellular calcium.^[26–28]^ Enhanced levels of reactive oxygen species are associated with cardiac remodeling, reduced left ventricle/ventricular (LV) function, and contractive dysfunction.^[29,30]^

Nitric oxide induces oxidative stress and is synthesized by nitric oxide synthase in many cell types, including cardiac myocytes. Both inflammation and oxidative stress induce sepsis. Inducible nitric oxide synthase is not constitutively active. When highly expressed, inducible nitric oxide synthase is vital in vasodilation and hypotension during shock.^[16]^ Its overexpression occurs in immune and myocardial cells and has adverse effects on the contractile function of myocardial cells, partly through the induction of reactive oxygen species, downregulation of adrenaline receptors, and decreased sensitivity to calcium.^[31]^

Patients with sepsis exhibit elevated serum prostanoid levels, such as thromboxane and prostacyclin. Prostanoids can alter coronary endothelial function, while cyclooxygenase inhibitors reduce prostanoid levels. However, no beneficial effect of cyclooxygenase inhibitor treatment on coronary microvascular homeostasis in sepsis has been observed.^[32]^

Endothelin-1 is a vasoconstrictor that may play a role in decreased cardiac function in patients with severe sepsis.^[33]^ However, only a few studies have examined its role in septic cardiomyopathy.

Mitochondrial dysfunction may be a causative agent of septic cardiomyopathy.^[14]^ Cardiomyocytes are rich in mitochondria, allowing them to produce energy in the form of adenosine triphosphate (ATP) and regulate intracellular calcium.^[34]^ Abundant ATP maintains the contraction and diastolic function of the heart; therefore, serious mitochondrial dysfunction is deleterious to the heart and plays a role in septic cardiomyopathy.^[35]^ The endoplasmic reticulum serves as an internal calcium store to support cardiomyocyte contractility and relaxation functions via tight recycling of cytoplasmic calcium.^[36]^ Recent studies have suggested that endoplasmic reticulum stress may play a role in septic cardiomyopathy; however, the mechanism by which it leads to cardiac dysfunction remains unknown.^[37,38]^

Dysregulation of adrenergic pathways in cardiomyocytes is a direct mechanism of cardiac depression in sepsis.^[2]^ Other mechanisms of septic cardiomyopathy include noncoding RNA, complement damage-associated molecular patterns, and pathogen-associated molecular patterns.^[2,14]^

### 3.2. Mechanistic changes to the heart underlying septic cardiomyopathy

Similar to other organs, heart dysfunction in sepsis is caused by a dysregulated host response to infection.^[1,34]^ Changes in cardiac function in septic cardiomyopathy are considered to be dynamic over time. Under normal conditions, cardiac output can meet the oxygen demand in peripheral tissues.^[16,39]^ In the early phase of sepsis, left ventricular ejection fraction (LVEF) is not impaired; however, stroke volume is low because of insufficient cardiac preload resulting from high vascular permeability and vasodilation.^[16,39,40]^ The heart rate may increase to compensate, although it is often insufficient to maintain adequate cardiac output.^[16,39]^ In severe sepsis, after increased fluid loading and permeability, stroke volume can recover, particularly in survivors, while LVEF temporarily decreases, partly because of a higher LV end-diastolic volume.^[16,39]^ Therefore, a low LVEF may indicate preload optimization and good adaptation.^[16,39]^ In the late phase of sepsis, despite being given more fluid than survivors, nonsurvivors have a lower LV end-diastolic volume, suggesting persistent hyperpermeability and preload deficiency.^[16,41]^ Hence, gaining an understanding of the different stages of septic cardiomyopathy can influence the timely consideration of various interventions.^[16,42]^

### 3.3. Changes to the heart in sepsis

Heart dysfunction in sepsis and septic shock is well known. The compensatory mechanism of the heart in sepsis is considered hyperdynamic, in which the cardiac output increases above the patient’s pretreatment background.^[13]^ Nonsurvivors of a septic cardiomyopathy episode maintained a normal ejection fraction and unchanged ventricular volume, whereas survivors had an ejection fraction below 40% and substantially increased mean end-systolic and end-diastolic ventricular volumes with normal stroke volume.^[43]^ This paradoxical finding may be explained by considering that, although LV systolic dysfunction should have resulted in decreased cardiac output, concurrent LV dilatation caused a preserved stroke volume if fluid resuscitation was adequate.^[43]^ Moreover, changes in the heart to maintain stroke volume include a reduction in LVEF and a transiently larger LV end-systolic or end-diastolic volume.^[39]^ Patients who survive a septic cardiomyopathy episode show a return to normal ejection fraction and ventricular stroke volume 7 to 10 days after the onset of sepsis.^[7,13]^

Many changes in the heart during sepsis can be detected using cardiac magnetic resonance imaging (MRI). For example, cardiac MRI can reveal ventricular dilatation, which reduces ventricular contractility and/or right ventricular (RV) and LV dysfunction with a reduced response to volume infusion.^[13,39,44]^ Additionally, altered metabolism and structural edema, rather than ischemia or infarction, can be observed.^[39]^ Similarly, cardiac MRI can indicate dilatation of the LV and global ventricular dysfunction without regional dysfunction.^[13]^

A recent study demonstrated that visual assessment of RV function using echocardiography in patients with septic shock revealed that RV dysfunction was associated with lower cardiac output, pulmonary artery pressure index, and RV stroke work index.^[40]^

Changes made by the heart to maintain its function during sepsis can negatively affect patients. For example, decreased cardiac contractility, mediated by enhanced nitric oxide production, may occur.^[13,45]^ In addition, there may be a decrease in the myofibril response to calcium, which induces mitochondrial dysfunction.^[11,13]^ Notably, the 1-year survival decreases when isolated RV dysfunction is observed in sepsis.^[11,40]^ Furthermore, myocardial edema may be associated with myocardial inflammation and functional impairment.^[46]^

## 4. Diagnosis of septic cardiomyopathy

Diagnosing septic cardiomyopathy can be difficult because of a lack of agreed-upon criteria. In addition, changes to the heart in sepsis can occur in multiple ways, such as LV or RV impairment during systole or diastole, and cardiac output and oxygen delivery to the peripheral tissues may or may not be adequate.^[13,39,40,44]^

The diagnosis of septic cardiomyopathy is usually based on echocardiography, biomarkers, and direct invasive and noninvasive measures of cardiac output.^[9,44]^

### 4.1. Echocardiography

Consensus and expert opinion state that every hemodynamically unstable patient should undergo echocardiography, which may assist in identifying septic cardiomyopathy.^[9,12,16,41,47]^ Echocardiography can aid in detecting LV and RV impairment.^[40,48]^ However, dependence on loading conditions is a limitation of echocardiography, particularly for LVEF. For instance, a low LVEF of < 50% during sepsis is not predictive of these patients.^[49]^ Newer techniques, such as speckle-tracking echocardiography, may aid in addressing this challenge. The parameters used to evaluate septic cardiomyopathy using echocardiography are listed in Table 1.

**Table 1:** Echocardiographic parameters for evaluating septic cardiomyopathy.

Parameter	Values suggestive of septic cardiomyopathy
LV systolic dysfunction^[50]^	Ejection fraction < 52% in men or < 54% in women S′ wave < 7.5 cm/s Global longitudinal strain < 20%
LV diastolic dysfunction^[50]^	Lateral E′ wave < 7 cm/s (septal < 10 cm/s) Lateral E/e′ ratio > 13 cm/s (septal > 15 cm/s)
RV dysfunction^[50]^	RV/LV dilatation > 0.6 TAPSE < 17 mm TDI Str′ wave < 10 cm/s RV fractional area change < 35%
Decreased cardiac output	Septic cardiomyopathy groups: severe 4.3 ± 0.3, moderate 7.5 ± 0.4, and mild 6.6 ± 0.5 L/min.^[51]^ The septic cardiomyopathy group was 3.4 ± 0.8 L/min, and the non-septic cardiomyopathy group was 5.5 ± 1.6 L/min.^[52]^ Septic cardiomyopathy group was 5.88 L/min, and the control group was 5.48 L/min.^[53]^

LV = left ventricular, RV = right ventricular, TAPSE = tricuspid annular plane systolic excursion, TDI = tissue doppler imaging.

Newer echocardiographic techniques, such as speckle-tracking echocardiography, involve the use of non-Doppler-based algorithms to track the displacement of acoustic speckles in the myocardium to measure changes in the lengths of myocardial segments.^[53]^ An advantage of speckle-tracking echocardiography is its less susceptibility to preload and afterload changes. A case-control study of patients with sepsis and septic shock reported that global longitudinal strain measurements obtained using speckle-tracking echocardiography revealed a greater degree of myocardial dysfunction in patients with septic shock, whereas LVEF did not differ between the groups.^[53]^ Another study reported that speckle-tracking electrocardiography could be used to detect early impaired cardiac function compared with LVEF using conventional echocardiography.^[54]^

Recent studies have found that LV dysfunction is common in patients with sepsis, and while RV dysfunction is less common, it is associated with short-term mortality.^[40,48,55]^ Similarly, RV dysfunction is associated with lethal arrhythmia and circulatory insufficiency independent of the LV systolic function.^[40]^ Notably, the visual assessment of RV dysfunction using echocardiography can be used to identify the risk of mortality, and correlates well with hemodynamic parameters monitored using a pulmonary artery catheter.^[40]^ In addition, echocardiographic assessments of RV function demonstrate better correlations with hemodynamic parameters than cardiac MRI assessments.^[40,56,57]^ Visual assessment of RV function using echocardiography is simpler than that using other indices, and it is rapid, noninvasive, and suitable for assessing hemodynamic parameters in patients with sepsis.^[58]^

### 4.2. Afterload-related cardiac performance

Echocardiography combined with hemodynamic monitoring indicators, such as cardiac output, cardiac index, and cardiac power index, can be used to assess the degree of heart dysfunction in sepsis. However, these parameters may overestimate heart function because reduced systemic vascular resistance can lead to normal cardiac output in patients with sepsis. Afterload-related cardiac performance is the ratio of the predicted to the measured cardiac output obtained using the indicator dilution method or a pulse contour analysis cardiac output monitoring device. A recent study identified afterload-related cardiac performance as a method for distinguishing septic cardiomyopathy from cardiovascular system failure.^[59]^ Afterload-related cardiac performance is a quantitative measure of septic cardiomyopathy that was first reported by Werdan et al^[60]^ who stated that afterload-related cardiac performance can reflect cardiac dysfunction at an early stage. The study indicated that afterload-related cardiac performance of ≤ 68.78% is an independent risk factor for 28-day mortality.^[59]^

### 4.3. Biomarkers

Biomarkers indicate myocardial dysfunction in sepsis.^[39]^ The most commonly studied biomarkers include troponin (elevated troponin is correlated with a greater degree of LV dysfunction), brain natriuretic peptides (which have great prognostic value in sepsis), histones, and heart-type fatty acid-binding proteins.^[9]^ Troponin is a specific indicator of myocardial damage, and its elevation is frequently observed in sepsis and septic shock.^[44]^ A meta-analysis showed that elevated troponin levels were associated with a higher risk of death in patients with sepsis.^[61]^ The ventricular myocardium releases brain natriuretic peptides in response to the stretching of the cardiac wall. One study found that increased brain natriuretic peptide levels were associated with septic cardiomyopathy; however, it also noted that LV filling pressures did not correlate with brain natriuretic peptide levels.^[62]^ A significant association between high circulating histone levels, new-onset LV dysfunction, and arrhythmias has been reported in patients with sepsis and no previous cardiac dysfunction.^[63]^ However, given that histones are inside the nucleus and can be released into the circulation owing to cellular death and excessive inflammation that occur during sepsis, determining whether the increase in circulating histone levels is the cause or result of septic cardiomyopathy is challenging. Heart-type fatty acid-binding proteins are essential in fatty acid metabolism in cardiomyocytes^[64]^ and are upregulated in patients with severe sepsis.^[65]^ Although these biomarkers are not specific for diagnosing septic cardiomyopathy, they may be useful for gaining an understanding of improvement or worsening of cardiac injury when used to identify trends.^[9]^

### 4.4. Electrocardiography

No electrocardiographic findings have been used to diagnose septic cardiomyopathy. Sinus tachycardia and atrial fibrillation are the most common rhythms detected using electrocardiography in patients with sepsis.^[66]^ Although electrocardiographic findings are not diagnostic of septic cardiomyopathy, any abnormal electrocardiographic findings should prompt further cardiac evaluation.

### 4.5. Cardiac MRI

Cardiac MRI is used for noninvasive imaging of the heart. Recent findings from a small study that included critically ill, sedated, and ventilated patients suggested that cardiac MRI can be used to evaluate cardiac parameters and detect myocardial edema, inflammation, and diffuse or focal fibrosis.^[44]^ These findings indicate that cardiac MRI is safe and feasible in critically ill, sedated, and ventilated patients.^[44]^

### 4.6. Invasive devices

Invasive devices, such as pulmonary artery catheters, are not commonly used to diagnose septic cardiomyopathy because they do not provide a mortality benefit.^[67]^ Noninvasive methods, such as pulse contour analysis, pulse pressure variation, and stroke volume variation, are often used to predict fluid responsiveness, which helps in decision-making for patients with sepsis; however, further studies are needed to validate their diagnostic utility in septic cardiomyopathy.^[9]^

### 4.7. Myocardial pathology

While catheter and biotome myocardial biopsies are not routinely performed to obtain a definitive diagnosis of septic cardiomyopathy, they can be useful in excluding myocarditis as a differential diagnosis.^[68]^ Most myocarditis cases are viral in origin; however, a small percentage of these cases can be associated with bacteremia or sepsis.^[69]^ It is important to note that bacteremia or sepsis does not necessarily result in bacterial myocarditis. Diagnosis of bacterial myocarditis is difficult and is often confirmed through autopsy histology after the patient’s death, although in rare cases, the diagnosis can be made by myocardial biopsy while the patient is still alive.^[68,69]^

Bacterial myocarditis produces myocardial injury that is directly caused by the bacteria, whereas in the majority of septic myocarditis cases myocardial damage is caused by an inflammatory cytokine storm that is a secondary response to the bacteremia.^[70]^ The treatment of bacterial myocarditis differs from that of viral lymphocytic myocarditis, in which the use of common treatments such as steroid therapy can exacerbate the bacterial infection, making steroid therapy difficult in some situations.^[69]^ Our group has reported a case of bacterial myocarditis diagnosed by myocardial biopsy and autopsy tissue diagnosis.^[71]^ In addition, a case of bacterial myocarditis has been diagnosed by endomyocardial biopsy at autopsy.^[70]^

postmortem assessment of sepsis-related death can be carried out. The heart can be a target organ in sepsis, and its involvement often determines the development of septic cardiomyopathy. An examination of the heart that includes tissue biopsy for the purpose of conducting histopathology and cytopathology can aid in the diagnosis of septic cardiomyopathy. The process involves a combination of data obtained from macroscopic analysis, including heart biopsy, microscopic analysis, and microbial investigations.^[72]^

### 4.8. Differential diagnosis

Fulminant myocarditis is caused by a viral infection and progresses rapidly to severe heart failure and cardiogenic shock; its initial features may clinically resemble septic cardiomyopathy and vice versa. Both diseases can cause fulminant disease and often resolve within 7 to 10 days; however, the mortality for each disease can be high during this period. However, these diseases require different treatments. For example, fulminant myocarditis requires mechanical circulatory support, whereas septic cardiomyopathy is less common (see the Treatment of Septic Cardiomyopathy section below).^[73]^ Furthermore, myocarditis has a plethora of possible causes, some of which may require immunosuppressive therapies that are not indicated for septic cardiomyopathy. Septic cardiomyopathy may also be present concurrently with distributive shock, which requires treatment with fluid resuscitation and broad-spectrum antibiotics.^[9]^

Owing to divergent treatments, differentiating between these disease states through repeated echocardiography is essential, as load-dependent indices of ventricular function, such as cardiac index and ejection fraction, do not reflect intrinsic myocardial contractile function during sepsis.^[74]^ Initial diagnostic evaluation may not aid in differentiating between fulminant myocarditis and septic cardiomyopathy, as distinctive features of both diseases (e.g., diastolic dysfunction, although this is more prevalent in sepsis) may be not evident on echocardiography.^[75]^ Multiple case reports describe the possibility that the presence of influenza infection from the influenza A virus may be another potential indicator suggestive of fulminant myocarditis; however, viral antigens in these cases rarely result directly in myocardial damage, which is rather mediated via an immune response.^[76]^ However, there is considerable overlap between the 2 diseases, and limitations in the diagnostic evaluation of an individual patient should be recognized. Extracorporeal membrane oxygenation (ECMO) may be required in septic cardiomyopathy or fulminant myocarditis, and the need for ECMO alone cannot determine whether a patient has septic cardiomyopathy or fulminant myocarditis. As the patient’s treatment and sepsis progress, determining the treated disease becomes easier over time with greater likelihood.

Another differential diagnosis of septic cardiomyopathy is catecholamine- or stress-induced cardiomyopathy (Takotsubo cardiomyopathy); however, there is little clinical evidence to confirm this. Gross visual examination appears to be an option, given the distinctive visual form and motion of the heart resulting from catecholamine-induced vasoconstriction in patients with catecholamine-induced cardiomyopathy.^[9]^

## 5. Treatment of septic cardiomyopathy

### 5.1. Current treatment options

The treatment of septic cardiomyopathy should address the underlying infection, along with supportive care, such as fluid replacement, oxygen therapy, and medications to improve heart function. The initial treatment of sepsis focuses on early recognition, infection control, and optimization of hemodynamic parameters with fluid resuscitation and vasopressor therapy.^[77]^ This treatment strategy is considered the standard therapy for septic cardiomyopathy.^[77]^ Table 2 summarizes the commonly used treatments for septic cardiomyopathy, which, in general, are similar to those used for sepsis alone.^[13]^

**Table 2:** Current treatment for septic cardiomyopathy.

Evidence	Current therapies	Mechanism of action	Benefits	Limitations for treating septic cardiomyopathy	Definition or indication criteria for septic cardiomyopathy
Strong recommendation, strong quality of evidence^[78]^	Fluids^[9,12,78]^	Maintain systemic circulation by compensating for a lack of preload	Familiar, well-studied treatment in septic shock	Excessive preload may produce exacerbation of right ventricular dysfunction, resulting in low cardiac output	Acute and reversible within 7–10 days; Global biventricular dysfunction with reduced contractility; Left ventricular dilation; diminished response to fluid resuscitation and catecholamines; Absence of acute coronary syndrome as etiology^[9,12,78]^
Strong recommendation, moderate quality of evidence^[78]^	Vasopressors^[78]^	Vasoconstriction by alpha- or beta-adrenergic, vasopressin, or dopamine receptor stimulation	Familiar, well-studied treatment in septic shock	Excessive increase in afterload may cause cardiac dysfunction, especially with phenylephrine and vasopressin^[9]^	Septic shock should be considered as a diagnosis in patients with sepsis-associated organ dysfunction, particularly for those requiring vasopressor therapy^[9,78]^
Weak recommendation, low quality of evidence^[9,78]^	Inotropics^[9,78]^	Act on myocardial Ca^2+^ handling, increase contractility, and improve cardiac function	An effective treatment for septic shock, especially for septic cardiomyopathy	Some cases do not necessarily require inotropic drugs, and prolonged use may induce arrhythmias	The Surviving Sepsis Guidelines define sepsis as life-threatening organ dysfunction secondary to a dysregulated host response to infection^[9,78]^
Weak recommendation, low quality of evidence^[78]^	Beta-adrenergic sensitizers: levosimendan*^[9,12,78]^	Calcium sensitizer and potassium channel activator	Increases myocardial contractility, may improve hemodynamic parameters and cardiac function	A meta-analysis revealed that levosimendan treatment showed no evidence of survival benefit^[79]^	Septic cardiomyopathy is defined as a reversible left ventricular systolic dysfunction^[12]^ The adrenergic pathway is dysfunctional in septic cardiomyopathy, and levosimendan could have an advantage by acting outside of the adrenergic pathway, which has not been shown by clinical studies^[9,78,79]^
Weak recommendation, low quality of evidence that requires confirmation in clinical trials^[80]^	Beta-blockers^[9]^	Inhibit sympathetic nerve stimulation by blocking beta receptors	Often used in real-world clinical practice. It may be useful in cases in which there is difficulty in maintaining blood pressure and systemic circulation because of tachycardia. Used in situations of tachycardia (inappropriate sinus tachycardia and tachycardic atrial fibrillation) or lethal arrhythmias (sustained or non-sustained ventricular tachycardia and possibly ventricular fibrillation). Decreasing the pulse rate increases stroke volume, thereby increasing cardiac output	The inhibitory effect of beta-blockers on myocardial contraction may exacerbate decreased cardiac output when cardiac function is impaired; thus, caution should be exercised in its use	Septic cardiomyopathy is characterized by left and/or right ventricular impairment during systole or diastole, with or without inadequate cardiac output and oxygen delivery^[9,80]^
Weak support, low quality of evidence^[81]^	Antiplatelet agents: tirofiban, abciximab^[81]^	Inhibit platelet adhesion	Not a commonly used treatment	Prospective trials of antiplatelet therapy have been discouraging^[81]^	Almost all patients with sepsis have abnormalities in coagulation^[81]^
Strong support, high quality of evidence^[78]^	Mechanical support^[9,52,56]^ types of mechanical support include the intra-aortic balloon pump, venoarterial extracorporeal membrane oxygenation, and Impella^[1,50]^	Indicated when septic shock is complicated by cardiogenic shock	Useful for cardiogenic shock that is difficult to treat with inotropic drugs and/or infusions. Septic cardiomyopathy is often associated with temporary loss of cardiac function, and patients can often be weaned off mechanical support	Complications associated with mechanical support devices include bleeding, thrombosis, and exacerbation of infection	Septic cardiomyopathy defined as a decrease in left, right, or biventricular ejection fraction followed by a recovery of function over a period of days to weeks^[2,82]^ Cardiogenic shock defined as a persistently low blood pressure with evidence of end-organ hypoperfusion and inadequate response to fluid resuscitation^[83]^ Decreases in right ventricular ejection fraction can be detected with a simplified ellipsoid geometric method^[56]^

This table includes the current treatment options, mechanisms of action, benefits and limitations of each treatment, and each article’s definition or indication criteria for septic cardiomyopathy. The type of recommendation for each therapy (strong, medium, or weak) and quality of the evidence (high, moderate, or low) are also provided.

* Levosimendan is not available in Japan. It is available in other countries, where its positioning is similar to that of inotropic drugs.

Fluid treatment is the mainstay of therapy and is often initiated in the early stages of sepsis. Fluids can help maintain systemic circulation by increasing stroke volume to increase cardiac output. However, in septic cardiomyopathy, excessive fluid can cause RV dysfunction and low cardiac output.^[9,12,77]^ The Surviving Sepsis Campaign recommends using vasopressors, with norepinephrine and vasopressin or dopamine as the first and second choices, respectively, for selected patients.^[77]^ However, current treatment options, such as vasoactive substances and inotropes, may be suboptimal for treating septic cardiomyopathy. For example, vasoconstriction by alpha- or beta-adrenergic, vasopressin, or dopamine receptor stimulation is not an optimal treatment for septic cardiomyopathy because of the excessive increase in afterload that may cause cardiac dysfunction, particularly with phenylephrine and vasopressin.^[9]^ Inotropic drugs are an effective treatment for septic shock in septic cardiomyopathy; however, prolonged use may cause arrhythmias.^[9,79]^ In addition, the Surviving Sepsis Guidelines recommend inotropes such as dobutamine for treating septic cardiomyopathy; however, in patients with sepsis, dobutamine does not improve prognosis and may have adverse effects.^[84,85]^ Beta-adrenergic sensitizers, such as levosimendan, increase myocardial contractility and may improve hemodynamic parameters and cardiac function^[79]^; however, levosimendan currently has weak support, and the available evidence is of low quality.^[77,80]^ Beta-blockers may be useful when maintaining blood pressure and systemic circulation is challenging owing to tachycardia.^[9]^ However, their use requires confirmation through prospective clinical trials that include patients with cardiac dysfunction.^[9]^ A large prospective randomized trial by Morelli et al^[86]^ involving the use of esmolol to target a heart rate of 80 to 94 bpm reported increased stroke volume and reduced need for vasopressors in patients with septic shock but specifically excluded those with cardiac dysfunction. A short-acting beta-blockade with landiolol was combined with the calcium-sensitizer levosimendan in a case series of 3 patients with severe decompensated heart failure, improving cardiac function and normalizing stroke volume.^[87]^ These results indicate that improved hemodynamics require further validation in larger studies with a patient population of interest.

The challenge in determining treatment is that septic shock can be considered as abnormally distributive shock, whereas when septic cardiomyopathy is complicated, cardiogenic shock may occur. The focus of treatment is acute intervention because septic cardiomyopathy is a reversible condition; thus, improvement in cardiac function indicates that the underlying cause, sepsis, is treated.^[8,13]^ As stated earlier, patients with septic cardiomyopathy who survive the episode recover cardiac function within 7 to 10 days^[7,13]^; therefore, they may not require long-term cardiac treatment. Therefore, the management of acute heart failure exacerbations associated with cardiomyopathies other than septic cardiomyopathy may be similar to that of acute heart failure exacerbations; however, the treatment of septic cardiomyopathy differs from that of other cardiomyopathies, in which treatment is usually expected to be on a long-term basis.^[8]^

Similar to that for heart failure, the treatment for cardiomyopathy depends on the type of cardiomyopathy and on whether heart failure is chronic or acute. That said, as discussed in,^[6,88]^ treatment for cardiomyopathies, other than septic cardiomyopathy, often includes angiotensin-converting enzyme inhibitors, angiotensin receptor blockers, and diuretics. A marked difference between septic cardiomyopathy and cardiomyopathies other than septic cardiomyopathy is that septic cardiomyopathy is, in principle, a reversible condition that typically resolves within 7–10 days.^[7,13]^ In addition, septic cardiomyopathy is associated with RV dysfunction, along with peripheral vascular and intravascular depletion, all of which are usually not observed in other cardiomyopathies.^[7,13,40]^

Treatment for chronic heart failure in cardiomyopathies with reduced EF, typically dilated cardiomyopathy, and septic cardiomyopathy share some similarities and differences. The choice of therapy is highly individualized, given the severity of the disease. The use of angiotensin receptor neprilysin inhibitors, sodium glucose cotransporter 2 inhibitors, beta-blockers and mineralocorticoid receptor agonists is part of the treatment strategies for these conditions.^[89–92]^ Of note, these treatments are not typically used as a replacement for diuretics; rather, they are often used in conjunction with them. It is important to note that it is currently not known whether these treatments can modify the recovery process of septic cardiomyopathy, especially considering that it generally reverses within 7 to 10 days.

Regarding treatments for acute exacerbation of chronic heart failure in cardiomyopathies other than septic cardiomyopathy with reduced EF, the Nohria-Stevenson classification can provide guidance in the classification of patients with heart failure. Four distinct profiles are distinguished: “Warm-Dry, Cold-Dry, Warm-Wet, and Cold-Wet.”^[93]^ The systemic inflammation associated with sepsis causes peripheral vasodilation, the peripheral vasculature becomes Warm-Dry as long as intravascular fluid is maintained and peripheral circulation is maintained. The condition then changes to Cold-Dry when intravascular fluid is reduced, dehydration occurs because of increased vascular permeability, and peripheral circulatory failure progresses. In this case, the preload of the right heart is reduced, and therefore, fluid infusion is necessary. Warm-Wet, occurs when there is congestion and adequate peripheral perfusion. If left ventricular dysfunction (left heart failure) occurs, low cardiac output occurs, but pulmonary congestion may occur because of an increase in pulmonary artery wedge pressure, in which case the patient enters the Cold-Wet state. Or infusions are necessary for hypotension due to intravascular dehydration, and inotropic agents are necessary for impaired cardiac function. If right heart dysfunction (right heart failure) also occurs, both sides of the heart fail, and the Cold-Dry or Cold-Wet state is exacerbated depending on the degree of right heart preload. When septic shock is complicated by cardiogenic shock, mechanical circulatory support may be necessary. A recent study has found that the Stevenson classification in combination with echocardiographic assessment is useful to define the patient’s profile.^[93]^ Treatment strategies for septic cardiomyopathy need to take into consideration features such as right heart dysfunction, peripheral vasodilation, and intravascular dehydration, which are distinguishing factors compared with other types of cardiomyopathies.^[48,94]^ Hence, the treatment approaches for other types of cardiomyopathy, such as dilated cardiomyopathy and other nonseptic cardiomyopathies and heart failure, are different and cannot readily translate to septic cardiomyopathy.

Antiplatelet agents inhibit platelet adhesion; however, these agents are not commonly used to treat septic cardiomyopathy, and findings from prospective trials have been discouraging.^[81]^ Although phosphodiesterase III inhibitors, such as milrinone, may be useful in these patients, no studies are specific to septic cardiomyopathy. However, phosphodiesterase III inhibitors are a class of medications known as inodilators, and therefore should be used for hypotension with caution. A non-randomized trial in patients with septic shock reported that the combination of milrinone and metoprolol was associated with improvements in cardiac index and a higher stroke volume.^[95]^ Similarly, Barton et al^[96]^ explored the use of intravenous milrinone in a prospective study of pediatric patients with non-hyperdynamic septic shock, reporting improvement in cardiac index, stroke volume index, and oxygen delivery, with no adverse effects, 2 hours after dosing. A notable impediment to this approach is its inability to advise for renal impairment, which is common in many patients with sepsis.

Regarding non-drug treatments, mechanical support, such as an intra-aortic balloon pump (IABP), venoarterial ECMO (VA-ECMO), or an Impella device, can aid cardiac output. However, despite good-quality evidence,^[77]^ they are invasive and have a high risk of complications. The reversible pathophysiology of septic cardiomyopathy may be a good indication for VA-ECMO and Impella “in cases that are complicated by cardiogenic shock.” However, there is no evidence for its efficacy, and it is unclear whether Impella and VA-ECMO can impact the recovery process of septic cardiomyopathy. However, the impact of VA-ECMO and Impella devices on the recovery process of septic cardiomyopathy is unknown and requires further study. Inserting artificial devices such as the Impella carries risks, including the potential for infections such as bacteremia. In addition, bacteremia can lead to coagulation disorders and inflammation due to severe infection or trauma, and can result in systemic activation of the coagulation system.^[97]^ A recent study has examined the subphenotypes of sepsis-induced coagulopathy to determine whether these subtypes of sepsis-induced coagulopathy exhibit distinct responses to anticoagulant therapy.^[98]^ The findings suggest that the effects of heparin treatment vary among these subphenotypes. These findings can help to gain an understanding of the heterogeneity of sepsis-induced coagulopathy, and aid in identifying patients who would benefit from anticoagulation therapy. In the presence of bacteremia, inserting foreign material can lead to secondary colonization by bacteria, making the eradication of the bacteria more difficult. However, in critical situations, life-saving measures such as VA-ECMO may be necessary.^[13]^ In cases of complicated cardiogenic shock, these mechanical circulatory assist devices may have to be inserted and implanted even if the infection is not under control. Ideally, however, the infection should be (somewhat) under control. For this purpose, it would be useful to observe improved inflammatory values and negative blood culture tests to reduce any risk of exacerbation of infection associated with device use.^[97]^ The choice of appropriate device (type of device and duration of use) is crucial for successfully treating septic cardiomyopathy.^[13]^ However, in practice, the risks and benefits must be weighed in the decision-making process.

### 5.2. Potential treatment options

There are several promising novel treatment options for septic cardiomyopathy (Table 3); however, most have been studied in laboratory and animal models. Hence, a nuanced approach must be adopted when considering novel treatments, noting that a mild or moderate decrease in cardiac performance might be an adaptive rather than a pathological response.^[39,103]^

**Table 3:** Potential treatments for septic cardiomyopathy.

Novel therapies	Mechanism of action	Benefits	Limitations in the treatment being addressed
*Schistosoma japonicum*-produced cystatin^[10]^	A strong immunomodulatory protein with anti-inflammatory features via activation of M2 macrophage polarization	May alleviate excessive inflammation and protect against sepsis-induced cardiac dysfunction	The findings are from basic research using a mouse model of septic cardiomyopathy and have not been clinically applied. Data reveal a potential mechanism by which *Schistosoma japonicum*-produced cystatin (Sj-Cys) may protect the heart
Melatonin^[37]^	Repression of receptor-interacting protein kinase 3 may reverse changes in mitochondrial dynamics, reduce endoplasmic reticulum stress, and prevent cardiomyocyte skeleton disassembly	Effective at attenuating septic myocardial injury	The findings are from basic research using a mouse model of septic cardiomyopathy and have not yet been clinically applied. Data reveal a potential mechanism by which melatonin protects the heart
Melatonin^[38]^	May stabilize B-cell receptor-associated protein 31 via the extracellular signal-regulated kinase pathway to preserve cardiac function	Effective at attenuating septic myocardial injury	The findings are from basic research using a mouse model of septic cardiomyopathy and have not yet been clinically applied. Data reveal a potential mechanism by which melatonin protects the heart
Gene therapy^[47]^	Genetic modifications that occur during sepsis cause activation of stress transcription factors such as nuclear factor kappa B and activated protein 1, which then activate multiple genes that encode inflammatory cytokines and chemokines, leading to septic shock	Gene therapy could reduce the inflammation associated with sepsis and improve cardiac dysfunction	The findings are from basic research using animal models and have not been clinically applied Targeted therapy with low rates of adverse effects
Glucose-insulin-potassium therapy^[99,100]^	Provides metabolic support to improve myocardial perfusion and left ventricular function. Prevents ischemia-related metabolic abnormalities	Increases cardiac output and temporarily improves hemodynamic status	Further studies are required to elucidate the role of glucose-insulin-potassium in septic cardiomyopathy
Hyperinsulin therapy^[101,102]^		Maintains euglycemia in sepsis and septic shock. May improve myocardial function	Further studies are required before this treatment can be provided to patients with sepsis

This table provides information on potential treatments, their mechanisms of action, and the proposed benefits of each treatment.

Elucidating the mechanisms underlying septic cardiomyopathy can provide novel treatment options for the condition, particularly considering that the current treatments are insufficient.^[9]^ Treating septic cardiomyopathy is hindered by variability in its definition^[9,41]^; consequently, which medicine to use for which presentation is not well defined.^[39,41]^ In addition, data comparing treatments, such as vasopressors and inotropes, in patients with and without septic cardiomyopathy are lacking.^[2,8,12]^ There are inadequate data and clinical trials on septic cardiomyopathy, posing a challenge for clinicians in deciding the best therapeutic approach for their patients.^[2,8,12]^ Therefore, clinical trials that include long-term outcomes to monitor cardiovascular health after recovery from septic cardiomyopathy are needed.^[41]^

Several potential therapies are currently being investigated, particularly using animal models of septic cardiomyopathy. Recent studies have reported attractive results regarding this. *Schistosoma japonicum*-produced cystatin (Sj-Cys) is a potential therapeutic agent for preventing and treating septic cardiomyopathy. Sj-Cys could alleviate excessive inflammation and protect against sepsis-induced cardiac dysfunction in a mouse model of sepsis-induced myocardial injury.^[10]^ Similarly, melatonin has been investigated as a potential treatment,^[18,37,38]^ as evidenced in a study where a mouse model showed that melatonin is effective in attenuating septic myocardial injury.^[38]^ Furthermore, gene therapy is promising as a possible therapeutic agent for septic cardiomyopathy and may reduce the inflammation associated with sepsis, improving cardiac function.^[47]^ One study showed that glucose-insulin-potassium therapy provided short-term improvement in the hemodynamic status of patients with septic shock and myocardial dysfunction.^[99]^ Elevated glucose levels are common in patients with sepsis.^[101]^ Hyperinsulin therapy may be useful for maintaining euglycemia in sepsis and septic shock, suppressing the inflammatory response, and improving myocardial function.^[101,102]^ Recent studies aimed at evaluating the role of thrombomodulin (specifically, its lectin-like domain) in the development of septic cardiomyopathy in mouse models^[104]^ indicated the potential importance of this pathway in inflammation and, in the future, may provide insights into developing therapies.^[105]^ Targeting a different pathway in inflammation (extracellular signal-regulated kinase 1/2 expression), sivelestat sodium reduces the expression of inflammatory factors and improves cardiac function in rats with induced sepsis,^[106]^ which may also offer potential treatment sites for patients with septic cardiomyopathy.

### 5.3. Practice guidelines

With no established objective definition of septic cardiomyopathy, specific and generally accepted treatment guidelines are unavailable. Beyond general management with vasopressors, inotropes, and fluid resuscitation, data remain sparse and evidence is lacking. However, some indications for the most recommended treatments may be gleaned from an examination of the more general sepsis guidelines that have been established. These sepsis guidelines vary regarding the attention paid to septic cardiomyopathy, and the low levels of evidence for specific treatments complicate generalization.

In 2021, the Surviving Sepsis Campaign^[78]^ updated its guidelines on managing sepsis and septic shock. As noted above, for adults with septic shock and cardiac dysfunction with persistent hypoperfusion, these guidelines included a suggestion (a weak recommendation with low quality of evidence) to add dobutamine to norepinephrine or use epinephrine alone for inotropic therapy; a similar weak recommendation suggests against using levosimendan.

The Japanese Clinical Practice Guidelines for Management of Sepsis and Septic Shock 2020^[107]^ specify that there is insufficient evidence for the effects of assisted circulation, such as VA-ECMO and IABP, on cardiac dysfunction in septic shock, and their applications in this setting are under investigation. Hence, a small single-center study comprising 12 patients reported a 6-month survival rate of 75% in patients with septic cardiomyopathy treated with VA-ECMO.^[108]^ In addition, the Japanese guidelines specifically mention the improvement in outcomes with VA-ECMO in recent years owing to improved devices and staff proficiency.

The American Academy of Emergency Medicine provides clinical practice statements on lactate measurement (as an indicator of poor prognostic outcomes)^[109]^ and the preferred resuscitation fluid (balanced crystalloid solutions)^[110]^ for patients with sepsis and septic shock. Neither of these clinical practice statements specifically refers to septic cardiomyopathy but to patients with sepsis in general.

The United Kingdom’s National Institute for Health and Care Excellence offers guidelines for sepsis, which were most recently updated in 2017.^[111]^ These guidelines do not specifically address cardiac dysfunction, except for the guidelines on intravenous fluid therapy, which recommend adjusting fluid volumes for patients with cardiac disease.

## 6. Conclusions

Despite substantial efforts made in studying the pathophysiological mechanisms and diagnostic options, there is no uniform definition for septic cardiomyopathy. Clinicians face many challenges when considering treatment, and a standardized definition of the condition is of great value. The focus of treatment for septic cardiomyopathy is acute intervention, whereas the treatment for other cardiomyopathies is provided on a long-term basis. In addition, several treatments for cardiomyopathy are contraindicated because of the profound hypotension associated with sepsis. Clinicians should also note that the changes in septic cardiomyopathy are dynamic. There is a paucity of data and clinical trials on septic cardiomyopathy. Hence, a greater understanding of the mechanisms underlying septic cardiomyopathy may contribute to developing a unified definition of the condition and designing novel treatment options.

## Acknowledgments

The authors thank Ryota Morimoto, MD, PhD; Toru Kondo, MD, PhD; Shingo Kazama, MD, PhD; Yuki Kimura, MD, PhD; Yuichiro Koyama, MD; and Ryota Ito, MD for their useful discussions and helpful comments regarding the manuscript.

## Author contributions

**Conceptualization:** Hiroaki Hiraiwa.

**Data curation:** Hiroaki Hiraiwa.

**Investigation:** Hiroaki Hiraiwa, Daisuke Kasugai.

**Methodology:** Hiroaki Hiraiwa.

**Visualization:** Hiroaki Hiraiwa, Daisuke Kasugai.

**Writing – original draft:** Hiroaki Hiraiwa, Daisuke Kasugai.

**Writing – review & editing:** Hiroaki Hiraiwa, Daisuke Kasugai, Takahiro Okumura, Toyoaki Murohara.
